# Anti-fibrinolytic agents in post partum haemorrhage: a systematic review

**DOI:** 10.1186/1471-2393-9-29

**Published:** 2009-07-15

**Authors:** Pili Ferrer, Ian Roberts, Emma Sydenham, Karen Blackhall, Haleema Shakur

**Affiliations:** 1 London School of Hygiene and Tropical Medicine, Keppel Street, London WC1E 7HT, UK

## Abstract

**Background:**

Post partum haemorrhage is a leading cause of maternal death worldwide. It also contributes to maternal morbidity as women may require a hysterectomy to control bleeding, or may require a blood transfusion, which can transmit viral infections. Anti-fibrinolytic agents have been proposed as a treatment for post partum haemorrhage. We conducted a systematic review to assess the effectiveness and safety of anti-fibrinolytic agents in post partum bleeding.

**Methods:**

All randomised controlled trials of anti-fibrinolytic agents given for bleeding during the postpartum period were included in this review. We searched Medline, PubMed, EMBASE, Cochrane Central Register of Controlled trials, Web of Science, metaRegister of controlled trials, LILACS, Reproductive Health Library, African healthline, POPLINE, MedCarib, CINAHL, Clinicaltrials.gov and the reference lists of eligible trials. Two authors extracted data. Methodological quality was assessed by evaluating allocation concealment. The primary outcome was maternal mortality. Secondary outcomes were blood loss, blood transfusion, hysterectomy, mean haemoglobin concentration, thrombo-embolic events and other adverse effects.

**Results:**

We identified three randomised controlled trials involving 461 participants. The trials compared tranexamic acid with no treatment and reported blood loss after delivery. In all three trials, allocation concealment was either inadequate or unclear. The administration of tranexamic acid was associated with a reduction in blood loss of 92 millilitres (95%CI 76 to 109). The most frequently reported adverse effect of tranexamic acid was nausea, although the increase was easily compatible with the play of chance (RR 4.63, 95%CI 0.23 to 95.14).

**Conclusion:**

Tranexamic acid may reduce blood loss in post partum haemorrhage. However, the quality of the currently available evidence is poor. Adequately powered, high quality randomised controlled trials are needed.

## Background

Each year, worldwide, about 536,000 women die from causes related to pregnancy and childbirth. Almost all (99%) of the deaths are in low and middle income countries.[1] Postpartum haemorrhage is the most common cause of maternal death.[2] Of the 14 million women who have postpartum haemorrhage each year, 1–2% die, with an average interval from onset of bleeding to death of 2 to 4 hours.[2] Haemorrhage is also an important cause of maternal mortality in high income countries, accounting for about 13% of maternal deaths.[3]

Postpartum haemorrhage also contributes to hospital morbidity because patients may require a blood transfusion, which can transmit blood borne viral infections. Approximately 1% of women with spontaneous vaginal deliveries receive a blood transfusion, but the rate increases to about 5% for women with instrumental deliveries or caesarean sections.[4] The risk of infection from transfused blood is higher in countries unable to thoroughly screen the blood used for transfusion.[5] In high income countries, the risk of transfusion transmitted infections is lower, but adverse reactions related to blood transfusion are a common adverse event.[6]

The World Health Organization (WHO) defines postpartum haemorrhage as blood loss of 500 ml or more.[7] The diagnosis is based on a clinical estimate of blood loss. The WHO states that the loss of 500 ml of blood should be considered an alert, after which the health of the woman may be endangered.[7] In many parts of the world, the loss of 500 ml of blood can be a serious threat to health due to the high prevalence of severe anaemia. Severe anaemia is a common consequence of postpartum bleeding and affects about 11% of the 14 million women with postpartum haemorrhage each year.[8]

The main causes of postpartum haemorrhage are uterine atony, trauma to the genital tract during delivery and retained placenta.[9] Risk factors for postpartum haemorrhage include primiparity, prolonged or augmented labour, multiple births, polyhydramnious, anaesthesia, macrosomia, obesity, and placental abruption. Nevertheless, the majority of women with postpartum haemorrhage have low-risk pregnancies.[2] There is evidence from randomised controlled trials that prophylactic oxytocin can reduce the incidence of post partum bleeding (relative risk [RR] for blood loss greater than 500 ml = 0.50, 95% Confidence Interval [CI] 0.43 to 0.59).[10]

The treatment of postpartum haemorrhage may include drugs to increase uterine contractility, volume replacement for blood loss, and various surgical procedures including uterine compression sutures, arterial ligation, selective artery embolisation, intrauterine tamponade and hysterectomy.[11-13] The cumulative incidence of emergency hysterectomy varies between regions but is estimated at between 0.04 and 1.25% for all deliveries.[14]

Systemic anti-fibrinolytic agents are widely used in surgery to prevent clot breakdown (fibrinolysis) to reduce blood loss. A systematic review[15] of randomised controlled trials of anti-fibrinolytic agents in elective surgical patients identified 211 randomised controlled trials including 20,781 randomised participants. The results showed that aprotinin reduced the risk of blood transfusion by a relative 34% (RR 0.66, 95%CI 0.61 to 0.71) and tranexamic acid by a relative 39% (RR 0.61, 95%CI 0.54 to 0.69). In those requiring transfusion, aprotinin reduced the transfused blood volume by 1.1 units (95%CI 0.83 to 1.31) and tranexamic acid by 1.1 units (95%CI 0.64 to 1.59). A pooled analysis showed that anti-fibrinolytic agents reduce the need for re-operation due to bleeding (RR 0.52, 95%CI 0.40 to 0.69) and there was a non-significant reduction in the risk of death (RR 0.90, 95%CI 0.67 to 1.20) in the anti-fibrinolytic treated group. There was no evidence of increased risk of developing thrombotic events.

The 5^th ^Millennium Development Goal is to reduce maternal deaths by 75% by the year 2015.[16] To achieve this goal, a reduction in maternal mortality of at least 5.5% each year is necessary. As haemorrhage accounts for around 25% of maternal deaths, an effective treatment for the management of postpartum haemorrhage could contribute significantly to the goal of reducing maternal mortality. Anti-fibrinolytic agents might reduce the need for hysterectomy, reduce the risk of severe anaemia and avoid the need for blood transfusion.

This systematic review aimed to quantify the effectiveness and safety of anti-fibrinolytic agents in the prevention or treatment of postpartum bleeding.

## Methods

We sought to identify all randomised controlled trials of an anti-fibrinolytic agent (aprotinin, tranexamic acid and epsilon-aminocaproic acid) with placebo or no treatment. A randomised controlled trial was defined as a trial in which the participants were assigned to one of two (or more) interventions using random allocation, or some quasi-random method of allocation. Participants were women with any type of bleeding from the genital tract during the postpartum period. The following electronic databases were searched up to November 2008: MEDLINE, PubMed, EMBASE, Cochrane Central Register of Controlled Trials, Web of Science SCI/ISI, metaRegister of Controlled Trials, Reproductive Health Library, LILACS, African healthline, CINAHL, POPLINE, MedCarib and Clinicaltrials.gov. Our search strategy did not include terms to identify participants (such as pregnancy or postpartum) as we wanted to find all randomised controlled trials of the use of antifibrinolytic agents, and classify them by the type of participant, thus ensuring maximum sensitivity. No language restriction was applied. Reference lists from relevant trials were also scanned. The list of medical subheadings (MeSH) and textwords used in the search strategy can be found in [See Additional file 1].

The primary outcome measure was mortality. Secondary outcome measures were blood loss (mean and standard deviation); amount of blood or blood components transfused (both the proportion of participants receiving the transfusion and number of units received); the occurrence of postpartum haemorrhage; the need for conservative surgical procedures (B-Lynch and other brace sutures, artery ligation, selective embolisation of arteries, and intrauterine tamponade independently of the technique used); peripartum hysterectomy; mean postpartum haemoglobin concentration; thromboembolic events: deep vein thrombosis, pulmonary thromboembolism, stroke, myocardial infarct; and other adverse reactions.

Data were extracted on the following items: number of randomised participants, types of participants and interventions, method of allocation concealment, loss to follow-up (number and reasons), the use of blinding, and whether an intention-to-treat analysis was carried out. In the event of insufficient information in the published report, the authors were contacted for clarification.

Data extraction was conducted by two review authors using a standardised data extraction form. The authors were not blind to authorship, journal of publication, or results of the trials.

Allocation concealment was the main element used to assess the methodological quality of included trials. Allocation concealment was scored according to the scale used by Schulz 1995.[17]

• A = trials that have taken measures that ensure allocation concealment (central randomisation; serially numbered, opaque, sealed envelopes; or other description with convincing elements of concealment).

• B = trials in which allocation concealment is not reported at all or the authors report an approach that does not fall into one of the other categories.

• C = trials in which concealment is inadequate (such as alternation or reference to case record numbers or dates of birth).

For blood loss volume, the difference in means (expressed in millilitres) was calculated with 95%CI. For other adverse reactions the RR and 95%CI were calculated. The between trial heterogeneity was assessed with the I^2^statistic. Data were combined using a fixed-effects model.

All analyses were performed with Review Manager^© ^version 5 and STATA^© ^version 10.1 for Windows.[18,19]

## Results

A total of 8,925 records were identified in the search. The titles and abstracts were screened by two authors and the full text of all potentially relevant trial reports were retrieved and read in full. Three trials[20-22] were identified that met the inclusion criteria [see Additional file 2]. The three trials included a total of 461 participants of whom 235 were randomised to receive tranexamic acid and 226 were randomised to a control group.

Of the three included trials, two were of women who had caesarean section delivery (Gai 2004; Gohel 2007) and one was of women who had spontaneous vaginal delivery (Yang 2001).

In Gai 2004 and Gohel 2007, one dose of 1 gram tranexamic acid was administered intravenously 10 and 20 minutes, respectively, before incision. The trial by Yang 2001 compared four groups. One group (n = 94) received a single dose of 1 gram tranexamic acid by intravenous infusion (IV), another group (n = 92) received a single dose of 0.5 gram tranexamic acid IV, the third group (n = 92) received a single dose of 0.5 gram aminomethylbenzoic acid IV and the fourth group (n = 87) served as a control group. In this review, only a comparison of the group that received 1 gram of tranexamic acid versus control was included in the analysis.

The method used to generate the randomisation sequence was specified in all three trials. In Yang 2001 the sequence was generated by computer; in Gai 2004 a consecutive numbered chart was used (no further explanation was given); in Gohel 2007 'the rule of even and odds' was used. The explanation given by the authors was that all odd cases were taken up for tranexamic acid. Allocation concealment was not described in Gai 2004 or Yang 2001. Gohel 2007 reported allocation concealment was inadequate. According to the Shulz scale, Gai 2004 and Yang 2001 were scored B; Gohel 2007 was scored C.

Randomised participants were followed up for a period of two hours after childbirth in all three trials. For further information on characteristics of the included trials, [see Additional file 3].

The primary outcome for this review was mortality. There were no deaths in the Gohel 2007 trial and mortality was not reported in the trials by Gai 2004 and Yang 2001.

Gohel 2007 reported a lower incidence of postpartum haemorrhage (defined as ≥ 500 ml blood loss from placental delivery to two hours postpartum) in women receiving tranexamic acid (5/50) than in the control group (14/50). Gai 2004 reported a lower incidence of postpartum haemorrhage (defined as blood loss of ≥ 400 ml) in the group receiving tranexamic acid (22/91) than the control group (36/89). Yang 2001 reported a lower incidence of postpartum haemorrhage (defined as blood loss of ≥ 400 ml in women receiving tranexamic acid (6/94) than the control group (22/87). The pooled relative risk for investigator defined (as above) postpartum haemorrhage was RR = 0.44 (95%CI 0.31 to 0.64).

Gohel 2007 reported that no thrombotic events occurred, and that no participants required blood transfusion. Gai 2004 and Yang 2001 did not report thrombotic events.

**Figure 1:**
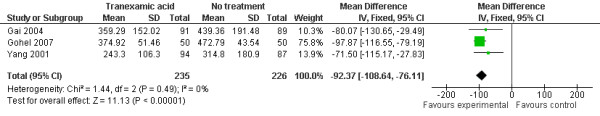
**Meta-analysis comparing tranexamic acid with no treatment**. Mean blood loss up to two hours after delivery, expressed in millilitres.

Combining the results of the three trials, the use of tranexamic acid significantly reduced mean blood loss by 92 millilitres (95%CI 76 to 109) compared to no treatment. There was no evidence of heterogeneity between trials (I^2 ^= 0%, Chi^2 ^= 1.44, degrees of freedom = 2, p = 0.49), [Figure 1].

Gohel 2007 reported no adverse reactions in any participant in the study. Gai 2004 reported transient mild adverse reactions, but the number of participants affected and the type of adverse reactions were not described. Adverse reactions were reported in Yang 2001; two participants in the intervention group developed nausea. Participants in the intervention group had a higher risk of nausea than participants in the control group (RR 4.63, 95% CI 0.23 to 95.1).

## Discussion

This systematic review and meta-analysis of three randomised controlled trials provides some evidence that a single dose of 1 gram of tranexamic acid given intravenously reduces mean blood loss within two hours of delivery. However, the quality of the included trials was poor and they provided no data on mortality, which was the primary outcome measure of the review.

The duration of follow up was short and adverse events may have occurred after the study period ended. Tranexamic acid is not completely eliminated from the blood until 9–18 hours after administration.[23] However, because the half-life of tranexamic acid is two hours, levels in the blood would be reduced after the study period. Additional outcome data collected beyond two hours of administration of tranexamic acid would have provided further information on adverse events.

The three included trials were all of low methodological quality. Selection, performance and detection bias may have influenced the results of the included trials. We cannot exclude performance and detection biases because participants in the control group did not receive a placebo. The reports did not mention whether or not the researchers were blind to allocation. As regards the meta-analysis of the effect of tranexamic acid on blood loss, the large standard deviations reported in the studies by Gai and Yang suggests that the data may be skewed, in which case our analysis of mean blood loss may be misleading.

Despite the importance of post partum haemorrhage as a cause of maternal mortality, there is little information from randomised controlled trials on the effects of antifibrinolytic agents as a treatment for this condition. Although our results are consistent with the results of randomised controlled trials of the use of tranexamic acid in surgical bleeding, showing a reduction in mean blood loss, the poor quality of the included trials warrants a cautious interpretation.

All of the trials included in this systematic review considered the use of tranexamic acid in the prevention of postpartum bleeding, and we found no trials of its use in the treatment of postpartum haemorrhage. Nevertheless, the recently updated postpartum haemorrhage treatment guidelines prepared by the WHO state that tranexamic acid may be used in the treatment of postpartum haemorrhage if other measures fail. However, the guidelines point out that the quality of evidence on which this recommendation is based is low and recommends that further clinical trials are conducted (personal communication from Dr Metin Gülmezoglu, Department of Reproductive Health and Research, World Health Organization, February 2009).

Although the cost of tranexamic acid is likely to vary by country and by producer, it is a simple and relatively inexpensive intervention. The time required to administer the treatment is short and no additional training is required. According to the British National Formulary, the cost of two 500 mg ampoules of TXA (Cyklokapron^® ^Pfizer) is three pounds sterling. If there were clinical benefits from using tranexamic acid, the benefits could easily justify the costs, whether in high, middle or low-income countries.

Although the results from this systematic review and the systematic review of the use of tranexamic acid in surgical bleeding are encouraging, a high quality randomised controlled trial comparing tranexamic acid to placebo is needed to determine whether tranexamic acid improves patient outcome in postpartum bleeding.

## Conclusion

In conclusion, there is insufficient evidence from randomised controlled trials to confirm or refute a clinically important treatment effect from tranexamic acid in post partum bleeding. The WOMAN trial is a large, international, randomised, placebo controlled trial of tranexamic acid in postpartum haemorrhage, and will provide this evidence .

## Competing interests

The authors are currently planning a large scale randomised controlled trial to evaluate the safety and effectiveness of tranexamic acid in the treatment of postpartum haemorrhage .

## Authors' contributions

IR and HS were responsible for the study concept and design. PF and KB developed the search strategy and ran the search. PF and ES identified relevant papers for inclusion and extracted data. PF performed the statistical analysis. PF, ES, IR and HS wrote the review. All authors read and approved the final manuscript.

## Pre-publication history

The pre-publication history for this paper can be accessed here:



## Supplementary Material

Additional file 1 **Search strategy**. Search strategy used to identify trials in electronic databases. Click here for file

Additional file 2 **Selection of studies**. Flow diagram of the selection of included trials. Click here for file

Additional file 3 **Characteristics of included trials**. Description of included trials. Click here for file
