# Supplementary material for: Anti-fibrinolytic agents in post partum haemorrhage: a systematic review
Source: BMC Pregnancy Childbirth. 2009 Jul 15;9:29. doi: 10.1186/1471-2393-9-29 (PMC2727491; doi:10.1186/1471-2393-9-29)
Supplement: Additional file 1 — Search strategy. Search strategy used to identify trials in electronic databases. [file 1471-2393-9-29-S1.doc]

| **MeSH terms** | 1. Antifibrinolytic-agents exp. 2. Aprotinin exp. 3. Aminocaproic Acids exp. 4. Tranexamic Acid exp. | |
| --- | --- | --- |
| **Textwords** | | 1.       Anti-fibrinolytic* or antifibrinolytic* or antifibrinolysin* or anti-fibrinolysin* or antiplasmin* or anti-plasmin* or (plasmin adj inhibitor*)).ab,ti.  2.       ((Basic or bovine or kunitz or kallikrein) adj1 (trypsin inhibitor* or trypsin inactivator*)).ab,ti.  3.       (Kallikrein-trypsin or bovine pancreatic trypsin or tranexamic or cyklokapron or pharmacia or t-amcha or amcha or ugurol or transamin or kabi or epsilon-aminocaproic acid or aminocaproic or lederle).ab,ti.  4.       (Amicar or caprocid or epsamon or epsikapron or aprotinin* or BPTI or antilysin or contrical or contrykal or dilmintal or iniprol or kontrikal or kontrykal or pulmin or trasylol or zymofren).ab,ti.  5.       ((Aminocaproic or 6-aminohexanoic or epsilon-aminocaproic) adj1 acid*).ti,ab.  6.       (Tranexamic acid or TXA or amcha or amca or cyklokapron or kabi-2161 or transamin or ugurol or t-amcha or trans-4-aminomethylcyclohexanecarboxylic acid).ab,ti.  7. (randomised or randomized or randomly OR random order or random sequence or random allocation or randomly allocated or at random or randomized controlled trial [pt] or controlled clinical trial [pt] or randomized controlled trials [mh]) and (Humans[mh]) |
