# Supplementary material for: Anti-fibrinolytic agents in post partum haemorrhage: a systematic review
Source: BMC Pregnancy Childbirth. 2009 Jul 15;9:29. doi: 10.1186/1471-2393-9-29 (PMC2727491; doi:10.1186/1471-2393-9-29)
Supplement: Additional file 2 — Selection of studies. Flow diagram of the selection of included trials. [file 1471-2393-9-29-S2.docx]

**Potentially relevant RCTs identified and screened for retrieval (n=8925)**

- MEDLINE (1950 to September Week 3 2008) 492 records
- PubMed (searched 01-10-08: added to Pubmed within last 90 days) 5 records
- EMBASE (1980 to Sept 2008) 2202 records
- Cochrane Central Register of Controlled Trials (*The Cochrane Library* Issue 3, 2008) 1819 records
- Web of Science SCI/ISI (1970 to 5 October 2008) 918 records
- metaRegister of Controlled Trials (6 October 2008) 77 records
- Reproductive Health Library (7 October 2008) 32 records
- LILACS (13 November 2008) 368 records
- African healthline (7 October 2008) 1074 records
- CINAHL (7 October 2008) 983 records
- POPLINE (7 October 2008) 759 records
- MedCarib (7 October 2008) 93 records
- Clinicaltrials.gov (6 October 2008) 103 records

**RCTs excluded (n=8913)**

- The trials were not randomised, did not use anti-fibrinolytic agents, involved participants who were not pregnant, etc.

**RCTs retrieved for more detailed evaluation (n=12)**

**RCTs excluded (n=9)**

- The trial participants were not pregnant.

**RCTs included in the meta-analysis (n=3)**
