# Supplementary material for: Anti-fibrinolytic agents in post partum haemorrhage: a systematic review
Source: BMC Pregnancy Childbirth. 2009 Jul 15;9:29. doi: 10.1186/1471-2393-9-29 (PMC2727491; doi:10.1186/1471-2393-9-29)
Supplement: Additional file 3 — Characteristics of included trials. Description of included trials. [file 1471-2393-9-29-S3.docx]

|  | **Methods** | **Participants** | **Interventions** | **Outcomes** | **Notes** | **Allocation Concealment** |
| --- | --- | --- | --- | --- | --- | --- |
| **Gai 2004** | Multi-centre RCT with unclear allocation concealment.  Randomisation was described as “180 primiparas were allocated to two groups using a randomized consecutive numbered chart”.  The blinding of participants, health care providers, outcome assessors and data analysts was not mentioned. | Term primipara with singleton delivered by caesarean section with attended regular perinatal care. | Intervention group: tranexamic acid, 1gm IV, 10 minutes before skin incision.  Both the intervention (n=91) and control (n=89) groups received oxytocin, 10 UI IV, and oxytocin, 20 UI intrauterine, after delivery. | Vital signs: heart rate, respiratory rate, blood pressure.  Postpartum blood loss up to 2 hours after delivery.  Incidence of postpartum haemorrhage, defined as >400ml blood loss.  Laboratory examinations including liver and renal function, and complete blood count.  Uterine contractility and placental separation.  Apgar score and birth weight.  Side effects of tranexamic acid. | Data were collected at three time points:  1. Before surgery.  2. Immediately after delivery of the placenta to the end of caesarean section surgery.  3. Two hours after delivery of the placenta. | B - Unclear |
| **Gohel 2007** | RCT with inadequate allocation concealment.  ‘Randomization was done by the rule of odds and even.’ Odd number participants received tranexamic acid.  Blinding was not done. | Primiparas and multiparas with singleton pregnancy, delivered by caesarean section. | Intervention group: tranexamic acid, 1gm IV, 20 minutes before skin incision. Both the intervention (n=50) and control (n=50) groups received oxytocin, 10 UI IV, and methylergometrine, 0.4mg, IV, after delivery. | Vital signs: heart rate, respiratory rate, blood pressure.  Postpartum blood loss up to 2 hours after delivery.  Incidence of postpartum haemorrhage defined as blood loss >500ml.  Adverse events from tranexamic acid for 2 hours postpartum. | Data were collected at four time points:  1. Before surgery.  2. Immediately after delivery of the placenta to the end of caesarean section surgery.  3. One hour after delivery of the placenta.  4. Two hours after delivery of the placenta. | C - Inadequate |
| **Yang 2001** | Multi-centre RCT with unclear allocation concealment.  Participants were ‘randomly assigned by computer into four groups’ during the second stage of labour.  Blinding was not mentioned. | Primipara with term singleton pregnancy, vertex presentation, and spontaneous delivery. | All participants received oxytocin, 10 UI IV, immediately after delivery of the fetal shoulders in the second stage of labour. Participants were then randomised into four groups.  Intervention Group I (n=94): tranexamic acid, 1 gm IV.  Intervention Group II (n=92): tranexamic acid, 0.5 gm IV.  Intervention Group III (n=92): aminomethylbenzoic 0.5 gm IV.  Control Group IV (n=87): no treatment. | Vital signs: heart rate, respiratory rate, blood pressure.  Postpartum blood loss up to 2 hours after delivery divided into two periods of time.  Incidence of postpartum haemorrhage, defined as >400ml blood loss.  Occurrence of adverse events within 2 hours after delivery. | Data were collected at two time points:  1. Immediately after delivery of the placenta.  2. Two hours after delivery of the placenta. | B - Unclear |
